# Longitudinal rural clerkships: increased likelihood of more remote rural medical practice following graduation

**DOI:** 10.1186/s12909-015-0332-3

**Published:** 2015-03-21

**Authors:** Denese E Playford, Asha Nicholson, Geoffrey J Riley, Ian B Puddey

**Affiliations:** 1 School of Primary, Aboriginal and Rural Health Care, Faculty of Medicine, Dentistry and Health Sciences, University of Western Australia, 35 Stirling Hwy, Crawley, WA 6009 Australia; 2 Faculty Office, Faculty of Medicine, Dentistry and Health Sciences, University of Western Australia, 35 Stirling Hwy, Crawley, WA 6009 Australia

## Abstract

**Background:**

Extended rural clerkships clearly increase the likelihood of rural practice post-graduation. What has not been determined is whether such rural interventions increase the likelihood of graduates practicing in more remote, versus inner regional, locations.

**Methods:**

The Australian Health Practitioner Regulation Agency database was used to identify the current workplace of every graduate of the Medical School of Western Australia, 1980 to 2011. There were 324 graduates working in a primary practice location defined by the Australian Standard Geographical Classification as inner regional to very remote. They were divided into 3 groups - 200 graduates who entered medical school before commencement of the Rural Clinical School of Western Australia (RCSWA), 63 who entered after the RCSWA had started, but not participated in RCSWA, and 61 who participated in the RCSWA. The RCSWA offers a longitudinal rural clinical clerkship throughout level 5 of the MBBS course.

**Results:**

The two groups not participating in the RCSWA had 45.5% and 52.4% of subjects in outer regional/very remote locations, respectively. In comparison, 78.7% of those who had participated in the RCSWA were currently practicing in outer regional/very remote locations. When the 3 groups were compared, the significant predictors of working in a more remote practice compared to working in an inner regional area were being female (OR 1.75 95% CI 1.13, 2.72, P = 0.013) and participating in the RCSWA (OR 4.42, 95% CI 2.26, 8.67, P < 0.001). In multivariate logistic regression that corrected for gender and remoteness of rural address before entry to medical school, participation in the RCSWA still predicted a more than 4-fold increase in the odds of practicing in a more remote area (OR 4.11, 95% CI 2.04, 8.30, P < 0.001).

**Conclusion:**

Extended rural clinical clerkship during an undergraduate MBBS course is related to a much greater likelihood of practicing in more remote, under-serviced rural locations.

## Background

Australia, like many other countries, suffers a chronic rural and remote doctor shortage [1] despite excellent urban supply [2]. Various initiatives aimed at correcting this disparity have been implemented, including opportunities for medical students to have prolonged rural clinical immersions through the establishment of Rural Clinical Schools [3]. The goal of these initiatives is primarily to increase the likelihood that medical graduates will work in rural and remote areas [4,5] but due to an approximate 10–15 year latency period between implementation of a rural training program within a medical school and ultimate practice by graduates of the new system, data on any resultant workforce location shifts are only just emerging [6,7].

Retrospective studies have shown a consistently positive relationship between rural training (especially longer term placements) and the likelihood of rural practice [8-10]. However, the effect of rural training has been difficult to disentangle from confounders such as the known positive relationship between a student’s rural background and the likelihood of rural practice upon graduation [8-10]. Self-selection effects also remain problematic as students who intend to practice rurally often choose to enter a rural training program, calling into question the effect of the training itself [11]. Early indications are that Rural Clinical Schools may be having an independent positive effect on urban-background students whose intentions without a Rural Clinical School experience are more likely to be for urban practice [7].

The relationship between rural training and remote practice is less clear. This is partly because the terms “rural” and “remote” are often ill-defined [12,13]. For example, the term “rural” has been used as a catch-all term for areas with low population density [14], and the terms “rural” and “remote” have been used interchangeably [15]. Others have used the term rural without any definition at all [9]. These inconsistencies indicate that by no means do the terms have standard meanings [16]. Where remote practice likelihood data exist distinctly from other regional data, studies of regional practice odds relative to remote practice odds have been insufficiently powered to show any meaningful differences [17]. However, it has been noted that postgraduate doctors who were vocationally trained at smaller sites were more likely to practice in rural areas and that these doctors tended to practice at sites similar to those in which they were trained [17]. It has also been shown that rurally trained undergraduates appear to prefer more remote locations as interns [18]. This suggests training delivered at remote sites should increase the likelihood of remote practice.

Some degree of order to this discussion can be brought by using Australia’s national remoteness classification system [19]. The Australian Standard Geographical Classification - Remoteness Area (ASGC-RA) describes five geographical areas based on the Accessibility/Remoteness Index of Australia (ARIA) [19]. ARIA is a continuous varying index with values ranging from 0 (high accessibility) to 15 (high remoteness), and is based on road distance measurements from over 12,000 populated localities to the nearest Service Centres in five size categories based on population size. Within this classification, ASGC-RA 1 refers to the Major Cities of Australia (ARIA score 0–0.2) which are characterized by relatively unrestricted accessibility to a wide range of goods and services and wide opportunities for social interaction. ASGC-RA 2 refers to Inner Regional Australia (ARIA score >0.2 and ≤2.4) and is characterized by some restrictions to accessibility of some goods, services and opportunities for social interaction. ASGC-RA 3 designates Outer Regional Australia (ARIA score >2.4 and ≤5.92) and is defined by significantly restricted accessibility, ASGC-RA 4 defines Remote Australia (ARIA score >5.92 and ≤ 10.53) with very restricted accessibility and ASGC-RA 5 defines Very Remote Australia (ARIA score >10.53) with very little accessibility.

Very little is known about the determinants of relatively remote practice (ASGC-RA 3–5) versus inner regional practice (ASGC-RA 2) among Australian rural doctors and whether it is influenced by rural training. The Rural Clinical School of Western Australia, a school in the University of Western Australia, offers a year of rural training in one of fourteen ASGC-RA 2–5 non-urban sites. This is an alternative to medical students obtaining the entire degree in an urban setting at the same university. In this paper we therefore explore the hypothesis that amongst graduates of the University of Western Australia who are currently working in rural practice, those who completed a longitudinal rural clerkship in the RCSWA are more likely to be practicing in more remote locations than graduates who completed all their training in urban areas.

## Methods

The study participants for this cohort study were drawn from all graduates of The University of Western Australia Medical School who entered from 1980 and had completed the course by 2011, ensuring that they were, at a minimum, in their third year after graduation. From February to May 2014, information was accessed from the Australian Health Practitioner Regulation Agency (AHPRA) database to identify each graduate’s current workplace location. Graduates were designated as working rurally if their primary practice location was in an area defined by the ASGC-RA as 2–5, and city/urban if ASGC-RA 1. Those identified as currently practicing rurally were the target population for this study. In terms of the reliability of the AHPRA database in identifying those currently practicing rurally, we have previously reported a more detailed comparison indicating at least 89% agreement against a corresponding RCSWA work location database [20].

### RCSWA participation

**Figure 1:**
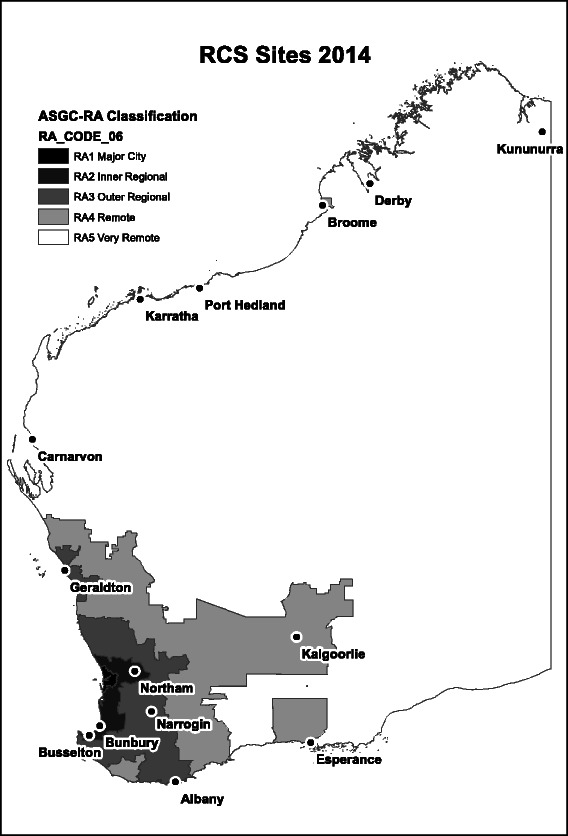
**ASGC-RA 2–5 Sites for the Rural Clinical School (RCS) of Western Australia (each site north of Carnarvon is currently defined as Remote, except for Derby which is defined as Very Remote).**

In 2002, the RCSWA commenced as a pilot project, with 7 volunteer students spending the second half of their 5^th^ MBBS year at one of 4 rural sites. In subsequent years, with the exception of international students, applications were invited from all students in the 4^th^ year of the course to spend the entire 5^th^ year of their course in the RCSWA. All applicants were subsequently ranked and 25% of each whole medical school cohort were then selected for participation, primarily on the basis of performance in a structured interview. From 2002 to 2010, the Rural Clinical School grew to 49 students over 12 separate rural sites. Figure 1 shows the current location of Rural Clinical School sites mapped to the ASGC-RA shape-file for Western Australia [20].

### Rural background

An active rural student recruitment process has been in place at the University of Western Australia since 2002 and by 2007 had resulted in the proportion of rural students increasing to a specified target of 25% of all students [21]. Our definition of “rural background” for those entering the Medical School at UWA has evolved over time. For students admitted from 1980 to 1998 no specific process was in place to define rural background students and so for the purposes of this study they have been defined as rural if they either had a rural correspondence address at entry to the course and/or completed secondary school in an area defined as ASGC RA 2 to 5. From 1999 to 2007 applicants were considered rural if they had lived in a rural area of Western Australia for a minimum of two years and, during that period, completed year 12 at a rural secondary school – “rural” being defined as a distance of >75kms from the Perth Central Business District. Students considered rural by these definitions were counted as rural in this study. Otherwise students were classified as “urban”. For all graduates included in the study, the ASGC-RA code for their town of origin prior to or at commencement of medical school was determined. For one rural background student this information was missing and so they were designated ASGC-RA 1.

### Study groups

All international students, who were ineligible for entry to the RCSWA, were excluded from the study. The final study cohort was linked to both a database of all students recruited from a rural background and to a database of all students enrolled in the RCSWA since its inception. It was divided into 3 groups for subsequent comparisons. The first group comprised those who had entered the university from 1980 onwards and who had from 1984 until 2001 completed the 5^th^ year of the MBBS course entirely in urban locations, prior to commencement of the RCSWA programme. The second group comprised all those who completed the 5^th^ year of the MBBS course after commencement of the RCSWA programme, but who likewise from 2002 until 2010 completed their 5^th^ year in urban locations. The third group also completed the 5^th^ year of the MBBS course from 2002 until 2010, but spent this year in the RCSWA. A comparison of current ASGC-RA practice location across these 3 groups permitted both an historical and a contemporaneous assessment of the impact of the RCSWA on the likelihood of more remote (ASGC-RA 3–5) versus inner regional practice (ASGC-RA 2) amongst those now practicing rurally.

### Socio-demographic factors

Age, gender and type of school attended - publicly funded government or fee paying independent - were recorded at entry to medical school. Age at graduation from medical school exhibited marked kurtosis - deviation from normal distribution - and skew and it was dichotomised into those 23 years and younger vs those 24 years and older. As a socioeconomic indicator, the correspondence postcode at entry for each student was linked to the Index of Relative Socioeconomic Advantage and Disadvantage (IRSAD) score from the Australian 2006 census Socio-Economic Indices for Areas (SEIFA) [22]. The construct for SEIFA codes, and the caveats in relation to their use as socio-economic indicators, have previously been described [21]. A substantial proportion of the MBBS cohort was born overseas but entered the course with Australian citizenship or permanent residency and were therefore eligible for the RCS. Region of origin for all students was determined from country of origin according to major regional groups as outlined in the Australian Standard Classification of Countries for Social Statistics [19]. Given the relatively small numbers of students in some groups they were collapsed into 5 groups for analysis - those from Oceania (Australia, New Zealand, Papua New Guinea and proximate Pacific islands), UK and Ireland, NE and SE Asia, Southern Asia (India, Pakistan, Sri Lanka and Bangladesh) and Other.

### Statistics

Data were analysed using IBM SPSS Statistics Release 20.0.0 (2011). All values are reported as Mean ± Standard Error of the Mean. Univariate comparisons of socio-demographic variables in each of the 3 study groups were made using the χ^2^ test. Univariate analysis of current practice in an ASGC-RA 3–5 location vs an ASGC-RA 2 location utilised logistic regression. A final multivariate logistic regression model was constructed for the major outcome variable of current site of practice in 2014 (ASGC-RA 2 versus ASGC-RA 3–5), using those socio-demographic variables identified as having significant predictive value in the univariate analyses together with rural background and participation in the RCSWA.

### Ethics

The project was approved by the University of Western Australia Human Research Ethics Committee, RA/4/1/1627.

## Results

Of the 3282 graduates of the University of Western Australia Medical School who entered from 1980 and had completed the course by 2011, 3020 were able to be tracked on the AHPRA register of medical practitioners between February and April of 2014, 5 were known to have deceased, 257 (7.8%) could not be found. Of the 3020 currently on the register, 108 (3.6%) were either overseas or registered as non-practicing, 2579 (85.4%) were in a city/urban site of practice and 333 (11.0%) were in a regional/rural site. From these, 9 international students were excluded leaving 324 subjects currently in rural practice as the final study cohort.

Of those currently working in a regional/rural site, there were 200 graduates who completed level 5 in medical school before 2002 and commencement of the RCSWA, 63 graduates who completed level 5 in medical school after commencement of the RCSWA but in urban locations and 61 graduates who also entered level 5 from 2002 but had participated in the RCSWA. Of the 61 participants in the RCSWA, 3 completed the RCSWA year at a site with an ASGC-RA index of 2 (4.9%), 24 at a site with an ASGC-RA index of 3 (39.3%), 32 at a site with an ASGC-RA index of 4 (52.5%) and 2 at a site with an ASGC-RA index of 5 (3.3%).

A comparison of each of the 3 study groups by socio-demographic factors at entry or exit from the medical school is outlined in Table 1. Since 2002, when the RCSWA commenced, there has been an increase in the number of females, an increase in age at completion of the course and as indicated by the ASGC-RA for town of origin, an increase in those from rural backgrounds, and also a significant trend towards recruitment of students from more remote areas. These characteristics were especially increased with respect to those who subsequently participated in the RCSWA. Recruitment of students from backgrounds of greater socioeconomic disadvantage was also evident, and at least in part, a consequence of the increased relative disadvantage seen in those from rural backgrounds.

**Table 1:** **Socio-demographic factors in the 3 study groups**

Variable	N	Pre RCSWA	N	Post RCSWA urban training	N	Post RCSWA rural training	P-Value (χ ^ 2 ^ test)
**Age at completion**							**<0.001**
Up to 23 yr	94	47.0%	12	19.0%	21	34.4%	
24 yr and older	106	53.0%	51	81.0%	40	65.6%	
**Sex**							**0.001**
Female	81	40.5%	34	54.0%	41	67.2%	
Male	119	59.5%	29	46.0%	20	32.8%	
**Secondary school type**							0.424
Government	77	53.1%	21	48.8%	18	41.9%	
Independent	68	46.9%	22	51.2%	25	58.1%	
**Secondary school location**							**<0.001**
Metropolitan	128	88.3%	32	74.4%	27	62.8%	
Rural	17	11.7%	11	25.6%	16	37.2%	
**IRSAD score**							**<0.001**
Decile 1-2	5	2.5%	1	1.6%	2	3.3%	
Decile 3-4	2	1.0%	2	3.2%	5	8.2%	
Decile 5-6	17	8.5%	5	7.9%	18	29.5%	
Decile 7-8	24	12.0%	15	23.8%	7	11.5%	
Decile 9-10	152	76.0%	40	63.5%	29	47.5%	
**Rural background**							**0.003**
Urban	165	82.5%	45	71.4%	38	62.3%	
Rural	35	17.5%	18	28.6%	23	37.7%	
**ASGC-RA Town of origin**							**0.004**
ASGC – RA 1 – Major City	166	83.0%	44	69.8%	36	59.0%	
ASGC – RA 2 – Inner regional	10	5.0%	8	12.7%	8	13.1%	
ASGC – RA 3 – Outer regional	14	7.0%	8	12.7%	13	21.3%	
ASGC – RA 4–5 – Remote/ Very remote	10	5.0%	3	4.8%	4	6.6%	
**Country of origin**							0.226
Oceania	103	63.6%	48	76.2%	50	82.0%	
UK and Ireland	16	9.9%	3	4.8%	2	3.3%	
Eastern and SE Asia	22	13.6%	5	7.9%	3	4.9%	
Southern Asia	4	2.5%	2	3.2%	2	3.3%	
Other	17	10.5%	5	7.9%	4	6.6%	

Significant P values are in bold-faced type.

**Figure 2:**
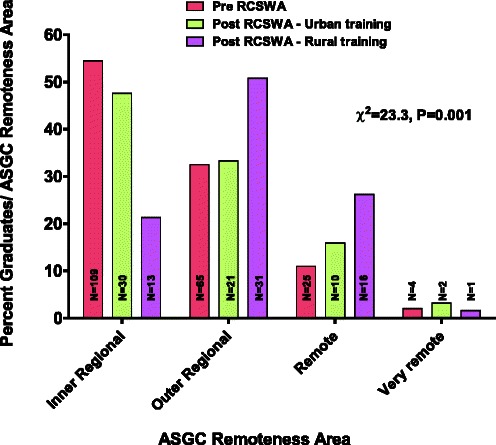
**Percent of graduates currently in regional/rural practice within each ASGC Remoteness Area for each of the 3 study groups.**

A breakdown of the ASGC Remoteness Area of current site of practice for each of these 3 groups is depicted in Figure 2 and Table 2. The pre-RCSWA group and post-RCSWA group who did not participate in the RCSWA show a similar distribution with 45.5% and 52.4% of subjects in ASGC Remoteness Areas 3–5, respectively. In comparison, for those who had participated in the RCSWA, 78.7% were currently practicing in ASGC Remoteness Areas 3–5. Univariate logistic regression indicated that participation in the RCSWA predicted a more than 4-fold increase in the odds of practicing in ASGC Remoteness Areas 3–5 (OR 4.42, 95% CI 2.26, 8.67, P < 0.001) compared to subjects in the pre-RCSWA group.

**Table 2:** **Univariate predictors of graduates currently in rural practice working in an area defined as ASGC-RA 3–5 vs ASGC-RA 2**

	Number (%) currently in ASGC 3–5 area	Odds ratio (Logistic regression)	P value
**Group**			
Pre RCSWA	91/200 (45.5%)	1.0	
Post RCSWA – Urban Training	33/63(52.4%)	1.32 (0.75, 2.32)	0.341
Post RCSWA – Rural Training	48/61 (78.7%)	4.42 (2.26, 8.67)	**<0.001**
**Age at completion**			
Up to 23 yr	68/127 (53.5%)	1.0	
24 yr and older	104/197 (52.8%)	0.97 (0.62, 1.52)	0.970
**Sex**			
Female	94/156 (60.3%)	1.75 (1.13, 2.72)	**0.013**
Male	78/168 (45.3%)	1.0	
**IRSAD Decile**			
1-2	5/8 (62.5%)	1.65 (0.39, 7.08)	0.499
3-4	5/9 (55.6%)	1.24 (0.32, 4.74)	0.754
5-6	25/40 (62.5%)	1.65 (0.83, 3.30)	0.155
7-8	26/46 (56.5%)	1.29 (0.68, 2.44)	0.438
9-10	111/221(50.2%)	1.0	
**Rural background**			
Urban	125/248 (50.4%)	1.0	
Rural	47/76 (61.8%)	1.51 (0.90, 2.56)	0.121
**ASGC-RA – Town of origin**			
ASGC – RA 1 – Major City	125/246 (50.8%)	1.0	
ASGC – RA 2 – Inner regional	12/26 (46.2%)	0.83 (0.37, 1.87)	0.652
ASGC – RA 3 – Outer regional	23/35 (65.7%)	1.86 (0.88, 3.89)	0.102
ASGC – RA 4–5 – Remote/Very remote	12/17 (70.6%)	2.32 (0.80, 6.79)	0.124
**Secondary school type**			
Independent	60/116 (51.7%)	1.0	
Government	69/115 (60.0%)	0.71 (0.42, 1.20)	0.206
**Secondary school location**			
Metropolitan	104/187 (55.6%)	1	0.885
Rural	25/44 (56.8%)	1.05 (0.54, 2.04)	
**Country of origin**			
Oceania	112/201 (55.7%)	1.0	
UK and Ireland	12/21 (57.1%)	1.06 (0.43, 2.63)	0.901
Eastern and SE Asia	11/30 (36.7%)	0.46 (0.21, 1.02)	0.055
Southern Asia	6/8 (75.0%)	2.38 (0.47, 12.10)	0.295
Other	14/26 (53.8%)	0.93 (0.41, 2.10)	0.927

Significant P values are in bold-faced type.

Each of the socio-demographic factors were also assessed in univariate logistic regression to determine the extent to which they might predict the likelihood of rural practice in ASGC-RA 3–5 vs ASGC-RA 2 (Table 2). The only significant predictor was being female (OR 1.75 95% CI 1.13, 2.72, P = 0.013). Multivariate logistic regression was undertaken forcing into the equation first both gender as well as ASGC-RA of town of origin prior to or at entry into the medical school. Participation in the RCSWA still increased the likelihood of rural practice in ASGC-RA 3–5 vs ASGC-RA 2 over 4-fold (OR 4.11, 95% CI 2.04, 8.30, P < 0.001) (Table 3).

**Table 3:** **Multivariate logistic regression with currently working in an area defined as ASGC-RA 3–5 vs an area defined as ASGC-RA 2 as the dependent variable and sex, ASGC-RA index for rural town of origin before entry to medical school and RCSWA participation as the predictor variables (N = 324) (Nagelkerke R Square = 0.112)**

Predictor variable	Intercept/B	Standard error	P value	Odds ratio	95% CI for odds ratio	
					Lower	Upper
**Sex**						
Female				1		
Male	0.360	0.236	0.128	1.43	0.90	2.28
**ASGC_RA – town of origin**						
ASGC – RA 1 – Major City				1		
ASGC – RA 2 – Inner regional	−0.489	0.444	0.270	0.61	0.26	1.46
ASGC – RA 3 – Outer regional	0.295	0.401	0.462	1.34	0.61	2.95
ASGC – RA 4–5 – Remote/Very remote	0.725	0.566	0.200	2.07	.68	6.26
**RCSWA participation**						
Pre RCSWA				1		
Post RCSWA – Urban Training	0.255	0.297	0.390	1.29	0.72	2.31
Post RCSWA – Rural Training	1.414	0.358	**<0.001**	4.11	2.04	8.30

Significant P values are in bold-faced type.

## Discussion

In this study we have been able to compare the rural practice distributions of all 324 entrants to our medical school from 1980 who had graduated by 2011 and who were currently in rural practice in 2014. Rurally practicing graduates who had completed the RCSWA were greater than 4-fold more likely to be in practice in an outer regional, remote or very remote location relative to inner regional practice. This four-fold increase was the case relative to rurally practicing graduates who had entered medical school before 2002 when the RCSWA was not in place. It was also the case relative to graduates of all backgrounds who had completed traditional discipline specific clerkships in an urban location after the RCSWA had started. So although this entire group was practicing rurally, the odds of being in practice in an outer regional, remote or very remote location was significantly higher for those who completed a longitudinal rural clerkship in the RCSWA.

It has been argued that the supply of doctors to remote locations is most likely to come from rural background students because rural background graduates enter rural work at higher rates [13,17]. However, we show that rural background was not related to higher odds of practicing in outer regional, remote or very remote locations relative to inner regional areas. Even coming from a more remote background at entry to the medical school was not a significant univariate predictor of likelihood of being in a more remote rural practice after graduation. So a strategy that only recruits rural-origin students is a less than optimal way to augment supply to remote areas of practice. To this end, the RCSWA intervention appears to be ultimately associated with a more remote workforce. This outcome may at least to some extent be a consequence of the fact that, given the vastness of the state of Western Australia and the relative remoteness of the majority of the RCSWA sites, the overwhelming proportion of RCSWA graduates spent a year at a rural site with an ASGC-RA 3–5 (10 sites) rather than an ASGC-RA of 2 (2 sites).

It could be argued that the RCSWA association with an ultimate choice to be part of a more remote medical workforce could be due to specific selection of students into the RCSWA who already intended to work both rurally and remotely. Although this could be the case, in an earlier study we showed that urban background female students, who would otherwise be expected to work urban at high rates, instead were working rurally at high rates after graduation from RCSWA [7]. This suggests some kind of change in intention, and is being followed up by further studies on students’ motivations on entry into medical school.

These data extend the results of a Canadian postgraduate study, which showed that graduates from regional and remote registrar training sites were more likely to be practicing both regionally and remotely relative to urban-trained graduates [17]. But that study did not further analyse differences between regional and remote work, did not partition these differences based on rural background, and did not take undergraduate experiences into account. Similarly an Australian study showed that relative to other graduates, interns from a regional university were more likely to be practising in an outer regional, ASGC-RA 3 area [20]. However as the number of graduates was small, there were no comparison data for more remote practice.

The finding of a greater likelihood of more females in remote areas of practice after participation in the RCSWA was a surprising outcome given previous literature outlining the many barriers to rural practice for women [23]. At least in part, our finding reflects the increasing proportion of females recruited in the course as well as the greater proportion of females applying for and being accepted into the RCSWA. It has been noted that the workforce proportion of women physicians is steadily increasing in many developed countries including Australia [24], but that female doctors are generally half as likely to be in rural practice [25,26]. Taken in isolation, these findings suggest the Australian “rural gap” will continue to grow [24]. However, in a US study it was shown that there was no difference in the likelihood of rural practice when female physicians participated in a rural clinical school training program compared to male graduates of the same program [26]. As these graduates in general had much higher odds of practicing rurally [12,26], this closed both the “gender gap” and the “rural gap” at once. Although data on women practitioners in Australia’s remote areas are scant [24], the above trends suggest that, as the proportion of female practitioners increases, the likelihood that they will choose to practice remotely will decrease [13] – except in the case of a rural clinical school training intervention.

We have carefully considered whether differences in socio-demographic profiles might have confounded our results. With respect to the changes in socio-demographic profiles before and after the commencement of the RCSWA, those observed in these rurally working graduates were similar to those we have previously published for all school leaver entrants to our medical school [21], namely an increase in the number of females and an increase in those with a rural background. Amongst the rurally based graduates who entered medical school with a rural background, there was also a trend towards increased recruitment of students from more remote areas and from areas of greater socio-economic disadvantage post commencement of the RCSWA. These changes therefore needed to be considered in relation to the simultaneous change in profile that was observed for relative remoteness of subsequent medical practice by those in rural practice in 2014. However, as already observed, coming from a more remote background at entry to the medical school was not a significant univariate predictor of likelihood of being in a more remote rural practice after graduation. Although female graduates were more likely than males to be in a more remote rural practice, when these 2 factors were taken into consideration in multivariate logistic regression, it was only participation in the RCSWA that remained a significant predictor of a still more than 4-fold increase in the odds of a more remote location of current rural practice. There was also a trend towards a higher age at completion of the course, more than likely related to the increased numbers of students taking a gap year after leaving secondary school and before commencing medical school, a trend more common amongst rural applicants. However, older age was not related to higher odds of remote practice. None of the potential socio-demographic confounders significantly attenuated the RCSWA effect on remote practice.

## Conclusion

For graduates who choose to practice rurally, having been immersed in regional and remote locations for one academic year as medical students, is associated with practice in more remote locations. In comparison, graduates currently practicing rurally but whose undergraduate clinical training was predominantly in urban locations are more likely to be in an inner regional practice. For underserved communities, this is an outcome with significant implications for increasing the medical workforce in more remote rural locations.
